# Evaluation of the prevalence of metachronous second primary malignancies in hypopharyngeal carcinoma and their effect on outcomes

**DOI:** 10.1002/cam4.4501

**Published:** 2022-01-26

**Authors:** Xi Luo, Xiaodong Huang, Shaoyan Liu, Xiaolei Wang, Jingwei Luo, Jianping Xiao, Kai Wang, Yuan Qu, Xuesong Chen, Ye Zhang, Jingbo Wang, Jianghu Zhang, Guozhen Xu, Li Gao, Runye Wu, Junlin Yi

**Affiliations:** ^1^ Department of Radiation Oncology National Cancer Center/National Clinical Research Center for Cancer/Cancer Hospital, Chinese Academy of Medical Sciences and Peking Union Medical College Beijing China; ^2^ Department of Head and Neck Surgical Oncology National Cancer Center/National Clinical Research Center for Cancer/Cancer Hospital Chinese Academy of Medical Sciences and Peking Union Medical College Beijing China

**Keywords:** hypopharyngeal cancer, incidence, metachronous second primary neoplasm, second primary neoplasm, survival analysis

## Abstract

**Background:**

To investigate the clinical characteristics of metachronous second primary malignancies (Met‐SPMs) and its impact on prognosis in hypopharyngeal carcinoma (HPC).

**Methods:**

We reviewed 593 newly diagnosed HPC patients without invasive synchronous SPMs (Syn‐SPMs) who were treated in our cancer center between 2009 and 2019. According to the status during follow‐up, patients were classified into three groups: (a) without SPMs (No‐SPMs, *n* = 440), (b) with tumors in situ in the esophagus or stomach (Tis, *n* = 80), or (c) with Met‐SPMs (*n* = 73).

**Results:**

The median follow‐up time for entire cohort (*n* = 593) was 66.7 months. Met‐SPMs were present in 12.3% of the cohort (73/593). The predominant site of SPMs was esophagus, followed by lung, oral cavity, thyroid, stomach, and oropharynx. In Met‐SPMs group, both index tumor and SPMs were the main causes of death. Tis group exhibited comparable 5‐year overall survival (OS) and disease‐specific survival (DSS) with that of No‐SPMs group. The Met‐SPMs group had similar 5‐year OS rate and better 5‐year DSS rate of 47.3% versus 43.6% (odds ratio [OR], 0.931; 95% confidence interval [CI], 0.681–1.274, *p* = 0.657) and 66.3% vs. 46.2% (OR, 0.600; 95% CI, 0.402–0.896, *p* = 0.012), respectively, compared with the No‐SPMs group.

**Conclusion:**

The overall incidence of Met‐SPMs in HPC was 12.3%. The occurrence of Met‐SPMs does not jeopardize the survival outcome of HPC. Routine surveillance of Met‐SPMs was requisite for patients with HPC.

## Graphical abstract

(1) Our study only focused on HPC as the index tumor; (2) Patients with Met‐SPMs achieved favorable outcome before and after PSM which shows these patients were high‐selected patients who had favorable index tumor control; (3) The routine surveillance and screening for Met‐SPMs are requisite for patients with HPC, and the UADT mucosa and the lung should receive more attention.

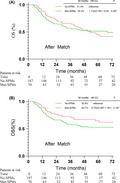

## 1 INTRODUCTION

Patients with head and neck squamous cell carcinoma (HNSCC) have a high incidence of second primary malignancies (SPMs) with a rate of 14%–36% reported in the literature.^1^, ^2^ Moreover, patients with an index tumor of hypopharyngeal carcinoma (HPC) presented even more frequent occurrence rate of SPMs.^3^, ^4^ Upper aerodigestive tract (UADT) is the most common risk region for developing SPMs, as the UADT mucosa is exposed to similar environmental carcinogens and triggering carcinogenesis at different sites.^5^ Besides, the development of cancer usually undergoes a succession of processes, including atypical hyperplasia, carcinoma in situ, and subsequent invasive carcinoma. Thus, different sites of the UADT mucosa may trigger carcinogenesis with various stages.

HPC features the worst prognoses among HNSCC, with the 5‐year overall (OS) rate of 30%.^6^ Advanced stage at initial diagnosis, occult early symptoms, and high recurrence of tumor have all been proposed as the causes of poor prognosis.^5^, ^7^ The high incidence of SPMs may compromise the prognosis.^5^, ^8^ Some studies have reported that patients with HNSCC with metachronous SPMs (Met‐SPMs) had a worse 5‐year OS rate (32%) than did patients without SPMs (69%).^9^ Conversely, others have reported that HNSCC patients with Met‐SPMs achieved even favorable outcomes.^10^

In the literature, most studies took all anatomic sites of head and neck together. However, HNSCC derived from different locations exhibited diverse biological behaviors and outcomes. The impact of Met‐SPMs on the survival of patients with index HPC may be underestimated or overestimated. In this regard, we mainly focused on Met‐SPMs in a large cohort of patients with HPC.

## 2 METHODS

### 2.1 Patient population

From April 2009 to October 2019 at our cancer center, we identified 593 patients with newly diagnosed HPC as the index tumor. All patients underwent comprehensive staging procedures. Endoscopic screening for UADT along with narrow band imaging (NBI) and Lugol chromoendoscopy (LCE) were routinely performed at our center. 96.0% of the patients (569/593) underwent oesophagogastroduodenoscopy before treatment. All these patients had no invasive synchronous SPMs (Syn‐SPMs) and received radical therapies. All patients were followed up regularly, every 3 months within 2 years, every 6 months for the following 3 years, and every year thereafter. Fibrolaryngoscope, oesophagogastroduodenoscopy, MRI, and CT of the head and neck, CT of chest was routine examination items. According to the follow‐up results, these patients were classified into three groups: (a) No‐SPMs (*n* = 440), (b) tumors in situ (Tis, *n* = 80), or (c) Met‐SPMs (*n* = 73). Patients in the No‐SPMs group had no SPMs during the follow‐up time. In the Tis group, patients developed Tis in the esophagus or stomach at initial diagnosis or during follow‐up. The Met‐SPMs group was defined as patients who developed invasive SPMs >6 months after the index tumor diagnosis.

The definition of multiple primary tumors (MPTs) was in accordance with the criteria of Warren and Gates and Hong et al.: (a) diagnosed as malignancy according to histological examination, (b) histologically distinct from the index tumor and metastasis was excluded, (c) at least 2 cm distance from the site of the index tumor or occurred at least 3 years after the diagnoses of the index tumor. Therefore, the SPMs are the first MPTs, the third primary malignancies (TPMs) are the second MPTs. Syn‐SPMs were defined as tumors that developed within 6 months of the index tumor diagnosis. Met‐SPMs were defined as those that developed >6 months after the index tumor diagnosis.

### 2.2 Data collection

All clinical data were obtained retrospectively from the patients’ clinical charts. The clinical stage of HPC was in accordance with the American Joint Committee on Cancer 8th edition (AJCC 8th). Extranodal extension (ENE) was evaluated with unambiguous evidence of gross ENE. The treatment strategies were divided into two groups: (a) primary S (patients underwent surgery first with or without postoperative radiotherapy) and (b) primary RT (patients received radiotherapy first with or without surgery).

Tobacco use was evaluated as the number of cigarettes/20 per day × number of smoking years (PY). Non, light, moderate, or heavy smoker was defined as never‐smoker, PY < 20, ≤20 PY ≤ 40, and >40 PY, respectively. Alcohol use was calculated based on the daily intake of ethanol: high, intermediate, and never/rare drinkers were defined as ≥60 g, 10–60 g, and ≤10 g ethanol intake per day, respectively. The body mass index (BMI) was calculated as the weight/height^2^.

### 2.3 Statistical analysis

We analyzed OS (defined as duration from treatment of index tumor to the last visit or death from any cause) and DSS (disease‐specific survival; defined as duration from treatment of index tumor to the last visit or death from HPC). We performed Kaplan–Meier survival analysis and compared differences with the log‐rank test. Categorical data are expressed as frequencies and percentages; differences between groups were tested using the chi‐square test. Prognostic factors were balanced between study arms by using propensity score matching (PSM).

This project was approved by the local Ethics Committee of the National Cancer Center (No. NCC3008) and was conducted in accordance with the Declaration of Helsinki. This article was reported in line with the STROBE (Strengthening the Reporting of Observational Studies in Epidemiology) guidelines for reporting observational studies.^11^

## 3 RESULTS

### 3.1 Clinical characteristics of index tumor and Met‐SPMs

A total of 593 patients with HPC as the index tumor received curative treatment from 2009 to 2019 at our cancer center. The baseline characteristics of the No‐SPMs group (*n* = 440), Tis group (*n* = 80), and Met‐SPMs group (*n* = 73) are shown in Table 1. There were no differences in terms of sex, age, ECOG (Eastern Cooperative Oncology Group) scores, BMI, proportion of ENE (+), treatment strategies, and smoking status among the three groups. However, the proportion of early clinical tumor‐node‐metastasis (TNM) stage (AJCC 8th) was higher in the Met‐SPMs group than in the Tis and No‐SPMs groups (*p* = 0.006). Alcohol intake were heavier in the Tis and Met‐SPMs groups compared with No‐SPMs group (*p* = 0.012).

**TABLE 1:** Baseline characteristics of patients

Characteristics	Total *N* = 593	No‐SPMs group *N* = 440	Tis group *N* = 80	Met‐SPMs group *N* = 73	*p*‐value
Age	12–83 (56)	12–83 (56)	36–73 (54)	34–80 (55)	
Sex					0.167
Male	573 (96.6)	422 (95.9)	80 (100.0)	71 (97.3)	
ECOG					0.469
0	43 (7.2)	36 (8.2)	3 (3.8)	4 (5.5)	
1	543 (91.6)	398 (90.4)	77 (96.2)	68 (93.1)	
≥2	7 (1.2)	6 (1.4)	0 (0)	1 (1.4)	
cStage (AJCC 8th)					0.006
I–II	34 (5.7)	19 (4.3)	4 (5.0)	11 (15.1)	
III–IVA	366 (61.8)	278 (63.2)	46 (57.5)	42 (57.5)	
IVB	193 (32.5)	143 (32.5)	30 (37.5)	20 (27.4)	
ENE					0.178
ENE (+)	146 (24.6)	111 (25.2)	23 (28.7)	12 (16.4)	
Treatment of HPC					0.399
Primary S	179 (30.2)	129 (29.3)	23 (28.7)	27 (37.0)	
Primary RT	414 (69.8)	311 (70.7)	57 (71.3)	46 (63.0)	
BMI					0.368
<18.5	50 (8.4)	34 (7.7)	10 (12.5)	6 (8.2)	
≥18.5	543 (91.6)	406 (92.3)	70 (87.5)	67 (91.8)	
Smoking					0.43
Non‐smoker	73 (12.3)	60 (13.6)	7 (8.8)	6 (8.2)	
Light (PY < 20)	164 (27.7)	118 (26.9)	28 (35.0)	18 (24.7)	
Moderate (≤20 PY ≤ 40)	229 (38.6)	170 (38.6)	30 (37.5)	29 (39.7)	
Heavy (>40 PY)	127 (21.4)	92 (20.9)	15 (18.7)	20 (27.4)	
Alcohol*					0.012
Never or rare	101 (17.0)	88 (20.0)	4 (5.0)	9 (12.3)	
Intermediate	182 (30.7)	132 (30.0)	25 (31.3)	25 (34.2)	
High	310 (52.3)	220 (50.0)	51 (63.7)	39 (53.5)	

Note

Data are *n* (%).

Abbreviations: BMI, body mass index; ENE, extranodal extension; HPC, hypopharyngeal carcinoma; Met‐SPMs, metachronous second primary malignancies; No‐SPMs, without second primary malignancies; Primary RT, received radiotherapy firstly; Primary S, received surgery firstly with/without postoperative radiotherapy; PY, pack‐years, number of cigarettes/20 per day × number of years of smoking; Tis, tumors in situ.

Alcohol*: We defined the following standard conversion ratios to assess ethanol consumption: 100 ml of liqueur was considered equivalent to 32.5 g of ethanol; 120 ml of wine to 11 g of ethanol; 633 ml of beer to 25 g of ethanol; high drinkers were defined as intake ethanol more than 60 grams per day; intermediate drinkers were defined as more than 10 grams but less than 60 grams per day; others were defined as never or rare drinkers.

The total incidence of HPC with invasive Met‐SPMs was 12.3% (73/593) and Tis in esophageal or stomach was detected in 13.5% patients (80/539). Among Tis group, 93.8% (75/80) occurred in esophageal and 6.2% (5/80) in stomach. The median interval between the Met‐SPMs and index tumor was 29.3 months (interquartile range [IQR] 22.0–47.3).

**FIGURE 1:**
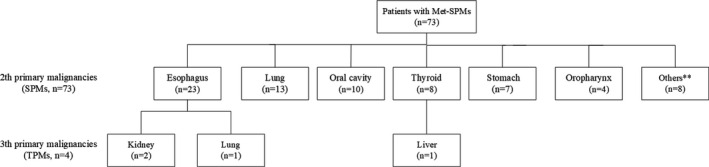
Distribution of multiple primary malignancies sites. Met‐SPMs, metachronous second primary malignancies; SPMs, second primary malignancies; TPMs, third primary malignancies. Others**: two located in centrum, one in larynx, one in urothelium, one in gallbladder, one in bile duct, one in liver and one in bladder

Esophagus was the most common site of the Met‐SPMs (31.5%, 23/73), followed by lung (17.8%, 13/73), oral cavity (13.7%, 10/73), thyroid (11.0%, 8/73), stomach (9.6%, 7/73), oropharynx (5.4%, 4/73), and other organs (11.0%, 8/73). Detailed distributions of the Met‐SPMs sites are shown in Figure 1. Moreover, four patients in the cohort developed a third primary tumor (TPT). In additional, more than half of the Met‐SPMs (57.5%, 42/73) harbored early stage of diseases (I‐II stage, AJCC 8th).

### 3.2 Prognosis of Met‐SPMs group

**FIGURE 2:**
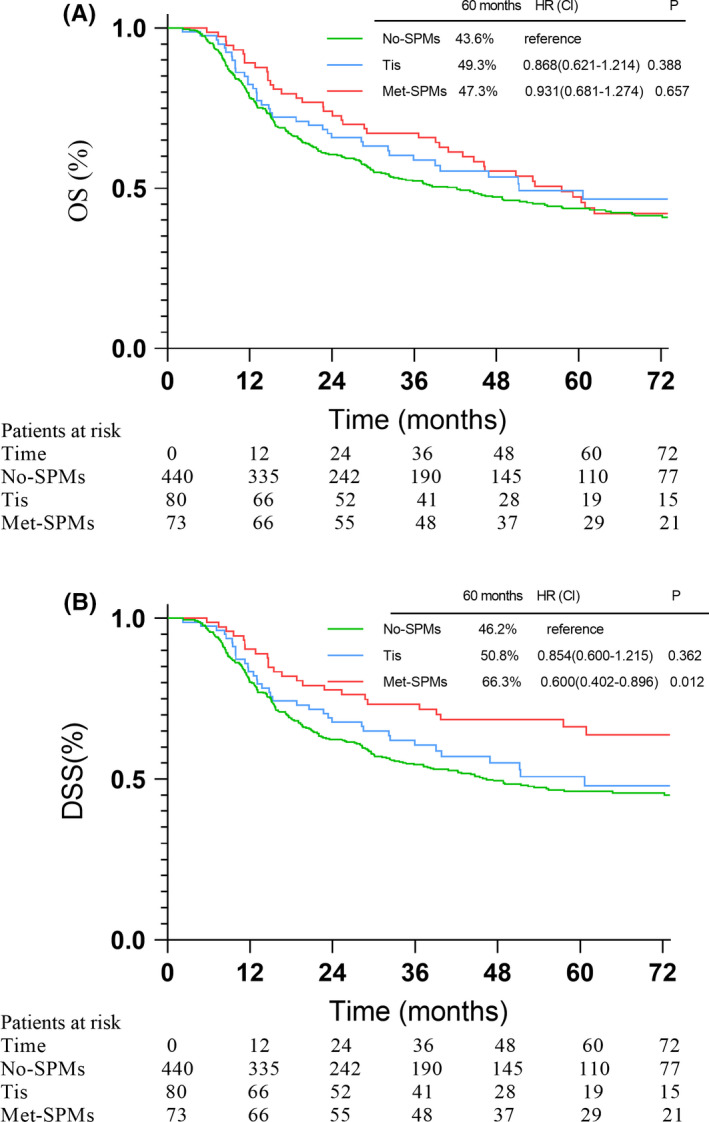
The overall survival (A) disease‐specific survival (B) among No‐SPMs group, Tis group and Met‐SPMs group

The median follow‐up time of entire cohort (*n* = 593) was 66.7 months (IQR 45.3–102.2), and were 66.6, 63.2, and 70.5 months for No‐SPMs, Tis, and Met‐SPMs groups, respectively. The 5‐year OS and DSS rates of the cohort were 44.6% and 49.4%, respectively. The OS and DSS rates of the No‐SPMs group (*n* = 440), Tis group (*n* = 80), and Met‐SPMs group (*n* = 73) are shown in Figure 2. Tis group achieved similar 5‐year OS and DSS rate as the No‐SPMs group: 49.3% versus 43.6% (*p* = 0.388) and 50.8% versus 46.2% (*p* = 0.362), respectively. The Met‐SPMs group shared similar 5‐year OS rate (47.3%) to No‐SPMs group (43.6%) (*p* = 0.657). The 5‐year DSS rate of the Met‐SPMs group was higher than that of the No‐SPMs group (66.3% vs. 46.2%, *p* = 0.012). OS and DSS comparisons between the Met‐SPMs and No‐SPMs groups are shown in Figure 2A,B.

### 3.3 PSM analysis of Met‐SPMs and No‐SPMs

**FIGURE 3:**
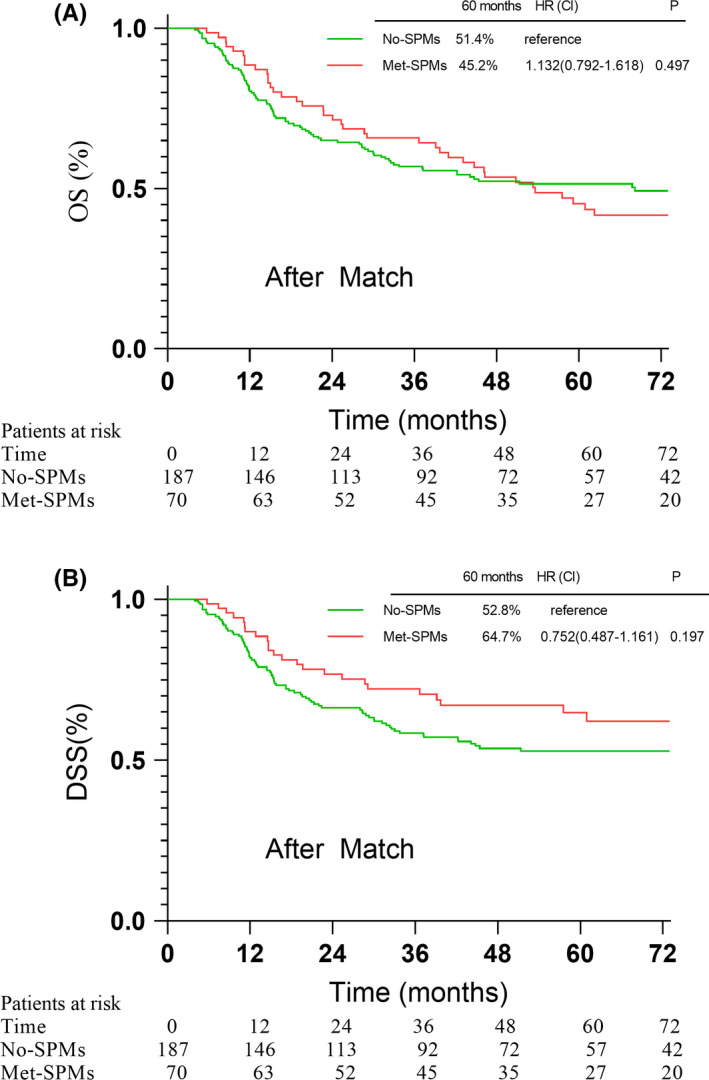
The overall survival (A) disease‐specific survival (B) between No‐SPMs group and Met‐SPMs group after PSM match

Baseline characteristics between Met‐SPMs and No‐SPMs groups were unbalanced. By using PSM method, two groups were balanced in terms of six prognosis factors with the including ratio of 1:3. After PSM, there were 187 patients in No‐SPMs group and 70 patients in Met‐SPMs group. The prognostic factors were well balanced between the two groups after PSM (Table 2). Adjusted 5‐year OS rate was 45.2% in the Met‐SPMs group and 51.4% in the No‐SPMs group (*p* = 0.497). The adjusted 5‐year DSS was 64.7% and 52.8% in Met‐SPMs group and No‐SPMs group (*p* = 0.197), respectively. The OS and DSS comparisons between the two groups before and after adjustment are shown in Figures 2 and 3.

**TABLE 2:** Clinical characteristics of index tumors for patients with metachronous second primary malignancies and without second primary malignancies

	Before PSM			After PSM		
	No‐SPMs	Met‐SPMs	*p*	No‐SPMs	Met‐SPMs	*p*
Total	440	73		187	70	
Sex			0.581			0.734
Male	422 (95.9)	71 (97.3)		183 (97.9)	68 (97.1)	
Age			0.042			0.845
>56	235 (53.4)	30 (41.1)		80 (42.8)	29 (41.4)	
≤56	205 (46.6)	43 (58.9)		107 (57.2)	41 (58.6)	
ECOG			0.727			0.959
0	36 (8.2)	4 (5.5)		11 (5.9)	4 (5.7)	
1	398 (90.4)	68 (93.1)		176 (94.1)	66 (94.3)	
2	6 (1.4)	1 (1.4)		0 (0)	0 (0)	
AJCC 8th			0.001			0.570
I–II	19 (4.3)	11 (15.1)		17 (9.1)	9 (12.9)	
III–IVA	278 (63.2)	42 (57.5)		121 (64.7)	41 (58.6)	
IVB	143 (32.5)	20 (27.4)		49 (26.2)	20 (28.5)	
ENE			0.103			0.914
ENE (+)	111 (25.2)	12 (16.4)		31 (16.6)	12 (17.1)	
Strategy			0.187			0.867
Primary S	129 (29.3)	27 (37.0)		70 (37.4)	27 (38.6)	
Primary RT	311 (70.7)	46 (63.0)		117 (62.6)	43 (61.4)	

Abbreviations: ENE, extranodal extension; Met‐SPMs, metachronous second primary malignancies; No‐SPMs, without second primary malignancies; Primary RT, received radiotherapy firstly; Primary S, received surgery firstly with/without postoperative radiotherapy.

### 3.4 Cause of death

Causes of death of the overall cohort three groups are demonstrated in Table 3.

**TABLE 3:** Cause of death for different groups

	Total (*n* = 593)	No‐SPMs group (*n* = 440)	Tis group (*n* = 80)	Met‐SPMs group (*n* = 73)
Total	327	240	40	47
HPC	86.9% (284/327)	92.1% (221/240)	90.0% (36/40)	57.4% (27/47)
SPMs	5.8% (19/327)	0 (0)	0 (0)	40.4% (19/47)
Others	7.3% (24/327)	7.9% (19/240)	10.0% (4/40)	2.2% (1/47)

Note

Data are *n* (%).

Abbreviations: Met‐SPMs group, metachronous second primary malignancies during follow‐up period; No‐SPMs group, without second primary malignancies during follow‐up period; Tis group, tumors in situ in esophagus or stomach when initial diagnosis or during follow‐up period.

During the follow‐up period, 327 patients (55.1%) died, with significant difference regarding the cause of death among groups (*p* < 0.001). In the Met‐SPMs group, HPC (57.4%, 27/47), and SPMs (40.4%, 19/47) were two main causes of death. However, HPC was the main cause of death in No‐SPMs and Tis groups.

## 4 DISCUSSION

The aim of our study was to describe the prevalence of metachronous second primary malignancies and its impact on prognosis of HPC as index primary in a large cohort. Our data shows that the total incidence of HPC with metachronous second primary malignancies was 12.3% (73/593) and Tis was detected in esophageal or stomach among 13.5% patients (80/593). Upper aerodigestive tract was the most common site that developed metachronous second primary malignancies. Prognosis was not influenced by Tis or metachronous second primary malignancies in patients with HPC.

Several studies have focused on second primary malignancies screening and distributions in HNSCC.^3^, ^10^, ^12^ In the present study, the total metachronous second primary malignancies incidence was 12.3%, which is consistent with previous Asian studies^3^, ^13^, ^14^, ^15^, ^16^ and is higher than that reported in European studies.^10^ For the involved sites in the present study, the esophagus was most predominant lesion, accounting for 93.8% (75/80) among patients with Tis and 31.5% (23/73) among patients with metachronous second primary malignancies. This was in line with the reports of other studies.^3^, ^5^, ^7^, ^17^, ^18^ The HN‐region (20.5%) and lung (17.8%) were also common involved organs of metachronous second primary malignancies. Similarly, Bugter et al.^10^ from the Netherlands reported that multiple primary tumors develop as frequently in the HN‐region (5.3%) as in the lung (4.9%). Others have observed that the stomach is another prevalent site of metachronous second primary malignancies.^3^, ^19^ Regarding the reason behind the organs involved, the HN‐region, esophagus, and lung are all upper aerodigestive tract mucosa and are exposed to similar environmental carcinogens which can be explained by the field cancerization theory.^20^

The 5‐year OS of No‐SPMs in our cohort was higher than the quoted historical result (43% vs. 30%),^6^ this may be explained by following reasons. The quoted historical result from a meta‐analysis included 87 randomized trials (16,485 patients) which conducted between 1965 and 2000, while our study included patients between 2009 and 2019. Due to advances in screening, radiology, surgical, radiotherapy technique, and chemotherapy regimen, the 5‐year survival of patients with HPC has improved over time. For one hand, conventional two‐dimensional RT (2D‐RT) and three‐dimensional conformal radiation therapy (3D‐CRT) was the standard technique before 2000, thus most of patients in historical meta‐analysis received 2D‐RT or 3D‐CRT. While all patients in our study were treated with modern advanced intensity‐modulated radiation therapy (IMRT). The advances in RT technique may contribute to the improved survival. For another hand, 199 patients (64.0%, 199/311) received concomitant chemoradiotherapy with standard cisplatin regimen, while in historical meta‐analysis, only 52% patients received CCRT with various regimens.

Prognosis was not influenced by metachronous second primary malignancies in patients with HPC. The Met‐SPMs group had similar 5‐year OS rate to the No‐SPMs group (47.3% vs. 43.6%, *p* = 0.657) before adjustment. In addition, the 5‐year DSS rate of the Met‐SPMs group was 66.3%, which was higher than the 46.2% in the No‐SPMs (*p* = 0.012, before adjustment). To exclude the impact of unbalanced prognostic factors, we balanced the two groups with propensity score matching method. After adjustment, the Met‐SPMs group presented a lower 5‐year OS rate (45.2%) compared with the 51.4% in the No‐SPMs group, albeit without a significant difference (*p* = 0.497). The 5‐year DSS rate of the Met‐SPMs group was 64.7%, which was numerically higher than the 52.8% in the No‐SPMs group whereas still without significant difference (*p* = 0.197). Our finding differs from those of some studies,^3^, ^19^ but is consistent with Bugter's study, in which 5‐year OS rate was 46.8% in patients with metachronous multiple primary tumors.^10^ This may be because of 1), the median interval between the Met‐SPMs and index tumor was 29.3 months (IQR 22.0–47.3), the long control time of the index tumor facilitated metachronous second primary malignancies development, patients without second primary malignancies had a high risk of locoregional failure within 2 years, with a 2‐year OS rate of only 40–60%.^21^, ^22^, ^23^ More than half of metachronous second primary malignancies (52.5%) were in early stage due to our regular follow‐up, which is easy to treat and so far not influence the prognosis of index tumor.

Many studies encourage routine surveillance and screening for metachronous second primary malignancies for head and neck cancer during follow‐up.^3^, ^5^, ^7^, ^17^, ^18^ Early diagnosis and positive treatment of the metachronous second primary malignancies may eliminate its negative impact on survival. Some studies have suggested that patients with early diagnosis of second primary malignancies may have a similar prognosis, as patients without second primary malignancies and with advanced diagnosis of second primary malignancies have much worse outcomes than patients without second primary malignancies.^24^ In the present study, the Tis group had similar prognosis to No‐SPMs group, with a 5‐year OS and DSS rates of 49.3% and 50.8% versus 43.6% (*p* = 0.338) and 46.2% (*p* = 0.362), respectively. Although more than half of metachronous second primary malignancies were in early stage (42/73, 57.5%), the metachronous second primary malignancies was the second cause of death in the Met‐SPMs group (40.4%). So far, regular and image‐enhanced endoscopy screening play an important role for detecting the metachronous second primary malignancies in early stage even in Tis.

In patients with HPC, the esophagus is the most common site for developing metachronous second primary malignancies, and several studies advocate routine surveillance and screening for esophageal second primary malignancies.^25^, ^26^ Some studies have even found that patients with early diagnosis and curative treatment of esophageal second primary malignancies may have a similar prognosis to patients without second primary malignancies. The HN‐region was the second common site of metachronous second primary malignancies and Bugter et al. reported a favorable prognosis among patients with HN‐SPMs^10^ (metachronous second primary malignancies in the HN‐region). Lung was the third most common region for metachronous second primary malignancies development, and advanced stage of lung second primary malignancies usually indicate low survival rates.^27^, ^28^ The interval between the metachronous second primary malignancies and index tumor was 29.3 months, routine endoscopic screening for the upper aerodigestive tract and CT or MRI for head and neck region and lung was strongly suggested for patients who have achieved long‐term locoregional control of primary HPC.

Our study has some limitations. The baseline of the index tumor between the No‐SPMs group and Met‐SPMs group was unbalanced. However, we circumvented this limitation with propensity score matching, and the numbers of patients were sufficient for comparison. Another limitation is that the number of different sites was small; we faced difficulty analyzing the survival of patients with metachronous second primary malignancies based on the different involved organs. Despite these limitations, the large total cohort size and our data collection made it possible to draw reliable conclusions.

## 5 CONCLUSION

The incidence of metachronous second primary malignancies in hypopharyngeal carcinoma patients is notable. The most common site of metachronous second primary malignancies was the upper aerodigestive tract. Prognosis was not influenced by metachronous second primary malignancies or Tis in patients with hypopharyngeal carcinoma. The interval between the metachronous second primary malignancies and index tumor was 29.3 months and more than half of metachronous second primary malignancies detected by regular and detail examination during follow‐up were in early stage. Similar outcomes in patients with Met‐SPMs compared to No‐SPMs may be driven by the combination of concurrent prolonged control of their primary hypopharyngeal carcinoma in this group and surveillance screening leading to second primary malignancies being diagnosed at early stages.

## CONFLICT OF INTEREST

None.

## AUTHOR CONTRIBUTIONS

Conception and design: Junlin Yi. Administrative support: Junlin Yi. Provision of study materials or patients: Junlin Yi, Runye Wu, Xi Luo, Shaoyan Liu, Xiaolei Wang, Xiaodong Huang, Jingwei Luo, Jianping Xiao, Kai Wang, Yuan Qu, Xuesong Chen, Ye Zhang, Jingbo Wang, Jianghu Zhang, Guozhen Xu, and Li Gao. Collection and assembly of data: Junlin Yi and Xi Luo. Data analysis and interpretation: Junlin Yi and Xi Luo. Manuscript writing: Junlin Yi and Xi Luo. Final approval of manuscript: All authors.

## Data Availability

Research data are stored in an institutional repository and will be shared upon request to the corresponding author.
